# Regional grafting of autologous adipose tissue is effective in inducing prompt healing of indolent digital ulcers in patients with systemic sclerosis: results of a monocentric randomized controlled study

**DOI:** 10.1186/s13075-018-1792-8

**Published:** 2019-01-07

**Authors:** Nicoletta Del Papa, Gabriele Di Luca, Romina Andracco, Eleonora Zaccara, Wanda Maglione, Francesca Pignataro, Antonina Minniti, Claudio Vitali

**Affiliations:** 1 U.O.C. Day Hospital Reumatologia, ASST G. Pini-CTO, Milan, Italy; 2 U.O.S. Chirurgia Vascolare, ASST G. Pini-CTO, Milan, Italy; 3 Sections of Rheumatology, Villa S. Giuseppe, Como and Casa di Cura di Lecco, Lecco, Italy

**Keywords:** Systemic sclerosis, Digital ulcers, Autologous fat grafting, Adipose tissue stem cells

## Abstract

**Background:**

A randomized controlled trial (RCT) was performed to confirm preliminary uncontrolled data indicating that regional adipose tissue (AT) grafting (G) is effective in inducing ischemic digital ulcer (IDU) healing in patients with systemic sclerosis (SSc).

**Patients and methods:**

SSc patients with IDUs were randomized to be blindly treated with AT-G or a sham procedure (SP). AT-G consisted of injection, at the base of the finger with the IDU, of 0.5–1 ml AT after centrifugation of fat aspirate. The SP consisted of false liposuction and local injection of saline solution. The primary endpoint was to compare the cumulative prevalence of healed IDUs in the two groups within the following 8 weeks.

**Results:**

AT-G and the SP were carried out in 25 and 13 patients, respectively. The two groups were comparable for age, gender, disease duration, and SSc subtypes. IDU healing was observed in 23/25 and 1/13 patients treated with AT-G and the SP, respectively (*p* < 0.0001). The 12 patients who received the unsuccessful SP underwent a rescue AT-G. In all of them, IDU healing was observed after 8 weeks of observation. It was noticeable that in the AT-G-treated patients a significant reduction of pain intensity (measured by visual analogue scale) was recorded after 4 and 8 weeks (*p* < 0.0001 in all cases). Similarly, a significant increase of capillary numbers in the affected finger was recorded by nailfold videocapillaroscopy after 4 and 8 weeks (*p* < 0.0001 in both cases).

**Conclusion:**

This RCT strongly confirms that AT-G is effective in inducing IDU healing in SSc patients.

**Trial registration:**

ClinicalTrials.gov, NCT03406988. Registered retrospectively on 25 January 2018.

## Introduction

Systemic sclerosis (SSc) is an autoimmune disease characterized by a multifactorial pathological process where a central role is played by the progressive loss of the microvascular bed, with the consequent fibrotic changes in the involved organs and tissues [1]. The clinical counterpart of the impaired microcirculation in the fingers is represented by Raynaud’s phenomenon, which is present in the great majority of the patients and is often the initial manifestation of the disease [2].

The progressive derangement of the microvascular architecture can be easily observed in finger periungueal areas by means of nailfold videocapillaroscopy (NVC). This simple and low-cost technique enables careful observation of the evolutionary steps of this pathological process. These steps are capillary network rarefaction, dilated or giant capillary formation, and, finally, almost complete vascular desertification [3]. The most advanced stages of capillary loss may induce the formation of digital ulcers (DUs) on the fingertips. This event, which has been observed in around half of the patients [2, 4, 5], commonly leads to a significant deterioration of the patient’s quality of life because of the often-severe disabling pain and the difficulty in performing the simplest daily living activities. The healing of DUs is often a lengthy process requiring accurate and intensive topical and systemic treatment [4]. Nevertheless, in a significant number of cases this therapeutic approach is ineffective and distal necrosis with subsequent tissue loss or phalangeal amputation may eventually occur.

Since it has been recognized that bone marrow mesenchymal stem cells (BM-MSCs) possess multiple regenerative and angiogenetic properties, therapy with these cells of autologous source has been tested with different modalities in different animal models and in some human pathological conditions characterized by peripheral ischemic lesions and wound formation [6], including SSc [7–9]. The positive results observed in these pilot studies have been ascribed to the capacity of BM-MSCs to secrete angiogenetic factors and differentiate themselves—as pluripotent cells—into various cellular types, all probably able to somehow support the neoangiogenesis [6].

Adipose-derived stromal cells (ASCs) are also believed to be pluripotent cells with characteristics similar to BM-MSCs. In addition, ASCs are regarded as a more favorable source since these cells can be easily and less invasively isolated, and have shown a great rapidity of expansion [10]. Preliminary attempts at cell therapy with ASCs have been carried out with the purpose of inducing ulcer healing in peripheral vascular impairment that can be observed in some animal models and human conditions [11]. In a recent open pilot study performed by our group, it has been demonstrated that grafting (G) with autologous adipose tissue (AT), which is known to contain both ASCs and a stromal/vascular fraction (SVF) [12], was effective in inducing prompt healing of long-lasting IDUs localized in the fingertips of a small number of patients with SSc [13]. The IDU healing was accompanied by the rapid disappearance of local ischemic pain and evidence of a partial restoration of the capillary bed in the digits when assessed by NVC.

Starting from these encouraging data, and with the purpose of confirming these preliminary results, we have designed and performed a monocentric randomized controlled study. In accordance with the study protocol, patients with a typical SSc-related IDU on the fingertip were randomized to undergo a regional AT-G with autologous fat as active therapy or a ‘sham’ procedure (SP)—that simulates the active treatment—as placebo treatment. All of the patients with SSc enrolled in both arms were blind regarding the treatment received. Furthermore, during the study period all of the enrolled patients received the same systemic vasoactive and topical therapy.

## Patients and methods

**Fig. 1:**
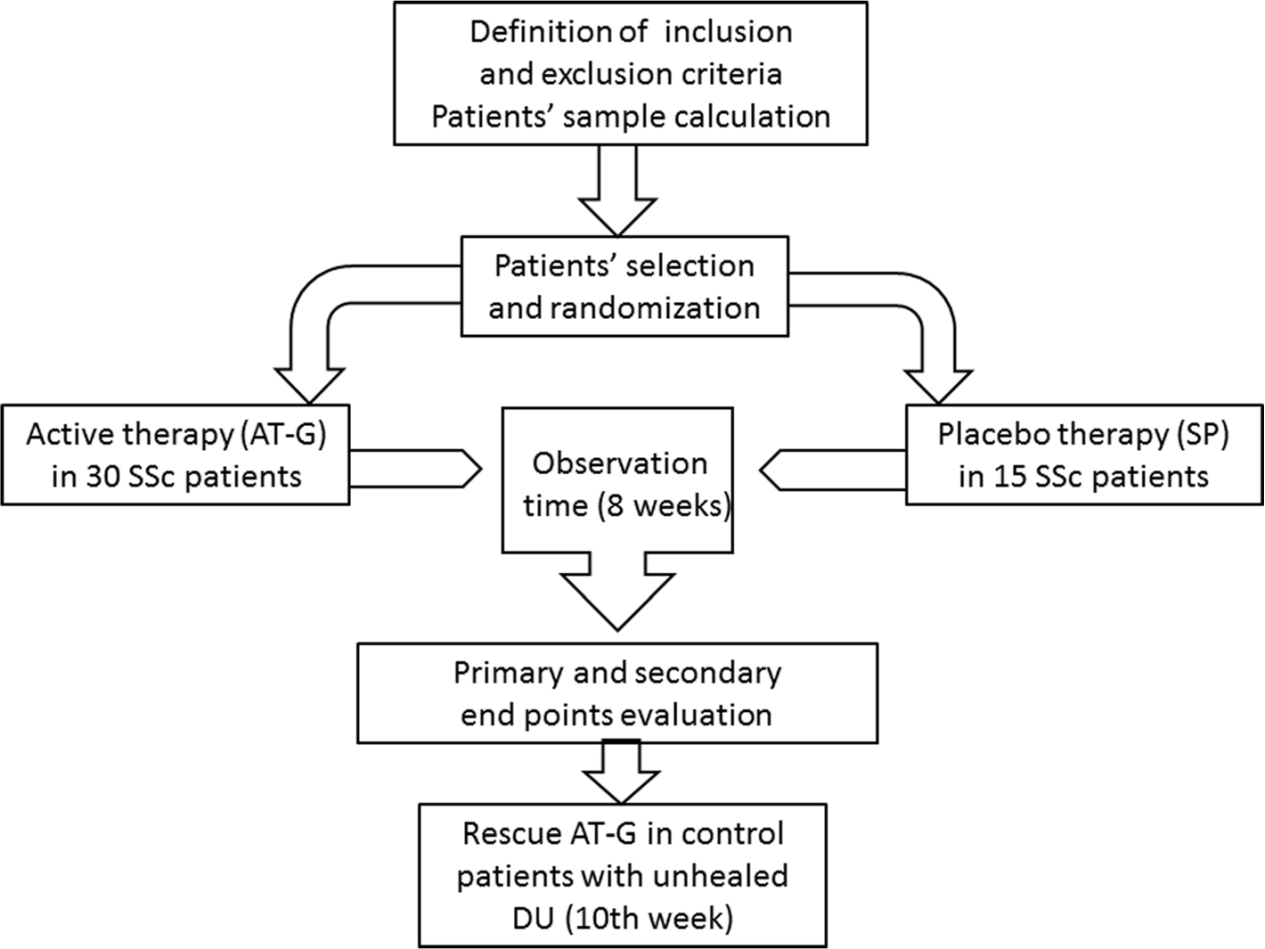
Flowchart for consecutive steps of the study. For more details, see Patients and methods. AT-G adipose tissue grafting, DU digital ulcer, SP sham procedure

The study design is schematically represented in Fig. 1. This study is registered with ClinicalTrials.gov (NCT03406988).

### Patients

The study was conducted from July 2017 to April 2018. Patients who were candidates for enrollment in the study were required to be over 18 years old and to meet the 2013 classification criteria of the American College of Rheumatology/European League Against Rheumatism for SSc [14]. Patients with either the limited cutaneous (lc) or the diffuse cutaneous (dc) variants of SSc were considered eligible for the study. Furthermore, all of the candidate patients had to have only one active IDU (cardinal ulcer), lasting for at least 6 weeks prior to enrolment and showing no tendency to heal despite weekly intravenous iloprost (0.5–2 ng/kg/min), oral administration of calcium-channel blockers (nifedipine 20 mg daily), local medication with meticulous attention directed toward wound care, including antimicrobial agents, keeping the DUs clean, and using a suitable dressing, and surgical removal of necrotic tissue. Previous clinical history of similar DUs did not preclude the eligibility of patients.

An IDU was defined as a painful area, at least 6 mm in diameter at its longest point, with visible depth and loss of dermis, located at the volar surface of the digit, distal to the proximal interphalangeal digital crease in a site judged compatible with a vascular etiology [15]. We excluded digital ulcers located over cutaneous calcifications or over extensor surfaces of small joints since they are generally believed to be mechanical, as a result of recurrent microtrauma and increased skin tension—although there is emerging evidence suggesting that all DUs in SSc could be the expression of the common vascular disease [16].

Exclusion criteria from the study were the presence of severe extracutaneous manifestations, such as cardiac, lung, and renal involvement, current therapy with dual and selective endothelin inhibitors, and concomitant treatment with immune-suppressive therapies (including prednisone equivalent > 10 mg). It is known that immunosuppressor agents have a cytostatic effect which could prevent regenerative processes and promote infections complicating DUs. Furthermore, there is insufficient evidence at present to recommend immunosuppressive therapy for DUs [17].

No patient included in the trial was in current therapy with phosphodiesterase type-5 inhibitors that are not authorized in Italy for the treatment of DUs.

Patients suffering from diabetes and/or other vascular diseases and women currently pregnant or breastfeeding were also excluded from the study recruitment.

### Active therapy

Active therapy consisted of the implantation of a small amount of autologous AT at the base of the finger where the IDU was distally located, following the procedure that was adopted in a previous open study [13]. Briefly, under local anesthesia, a total amount of 0.5–1 ml of the autologous AT was injected at the base of the affected finger, by sequential introduction of small aliquots in different directions from the injection site. This was done to spread the grafted AT as widely as possible around the finger base. “Luer lock” syringes (Artsana SpA, Grandate, Como, Italy) and Coleman 18G cannulas (Mentor-Aesthetic, Santa Barbara, CA, USA) were used for the procedure. At the moment of needle removal, a small skin incision was made at the site of injection to slightly reduce the tissue pressure due to the insertion of the additional material.

Autologous AT was previously harvested in each patient through liposuction from abdominal fat in the surgery room, under local anesthesia. Afterward, the collected AT was transferred into sterilized vials and processed at 920 × *g* for 3 min. The upper and lower phases, containing oil supernatant and mature adipocytes and blood and plasma residuals, respectively, were discharged. Only the intermediate layer was used for the filling procedure [13].

### Sham procedure

To maintain the blindness of the study, a SP was administered to the patients enrolled in the placebo group to simulate the AT-G. Briefly, a false liposuction of the abdominal adipose tissue was performed followed by the injection of 0.5–1 ml of 0.9% saline solution at the base of the affected finger. The SP was carried out exactly in the same surgical room and following the same steps as for the active therapy.

### Basal therapy

Weekly iloprost infusions and calcium-channel blockers that were administered to the patients before inclusion in the study were continued during the entire observation time for all of the patients enrolled in both arms of the study. The administration of analgesics to alleviate the IDU-related pain was also allowed for each patient. The amount of analgesics administered was obviously drastically reduced and stopped when pain disappeared as a consequence of IDU healing.

### Outcome parameters and follow-up of the patients

The primary endpoint of the study was to compare the prevalence of patients in whom IDU healing was observed within 8 weeks after the AT-G and to compare this figure with that observed in the control arm of the study where the SP was performed. To verify the time of IDU healing, all of the patients treated with either AT-G or the SP were observed every week. Local medication and necrotic tissue debridement were also performed during this weekly observation when required.

Secondary endpoints to be assessed were: pain improvement (i.e., a reduction of more than 50% of the baseline pain VAS score after AT-G and the SP); and variation of the number of capillaries in the affected digits in patients who received AT-G or the SP.

To this end, a patient’s self-assessment of pain severity was performed by means of a visual analogue scale (VAS) (range 0–100 mm, 100 mm indicating the most severe pain). This was done immediately before and every week after carrying out AT-G and the SP in all patients included in the study. Moreover, an NVC examination (with a 200× magnification lens, using Videocap; Scalar Co. Ltd, DS MediGroup, Milan, Italy) was performed in every affected finger immediately before and after 4 and 8 weeks for every patient enrolled and randomized to receive both types of procedure. NVC images from each patient were taken from four consecutive fields, two fields in both the right and left directions, starting from the middle of the nailfold, for a total extension of 1.2 mm and digitally stored. Although all studied patients exhibited a capillaroscopic late pattern according to Cutolo et al.’s classification [3], we decided to evaluate only the count of capillaries, since it was easier to precisely quantify this parameter than the number and extension of avascular areas. A single experienced operator, who was blinded regarding the type of procedure performed in the examined patient, counted the cumulative number of observed capillaries before and 4 and 8 weeks after both AT-G and the SP.

### Rescue AT-G therapy

The follow-up period was extended beyond the 8 weeks when the primary and secondary endpoints were assessed up to a total of 20 weeks. It was preliminarily established that all of the patients in the control arm, in whom IDU healing was not observed after 10 weeks, had to undergo a rescue AT-G. The possibility that IDU healing could be achieved after this rescue therapy was verified by monitoring these patients every week for the following 10 weeks.

### Calculation of the patients’ samples and randomization procedure

Starting from the results of our open study that showed 100% of IDU healing within 10 weeks in the patients treated with AT-G, and from some literature data demonstrating that, in patients suffering from distal IDUs and treated with standard vasoactive therapy, ulcer healing was observed in around 15% after 9 weeks [18], we have considered it plausible to achieve IDU healing after 8 weeks of observation in at least 80% of patients treated with AT-G and in no more than 30% of the control patients receiving the SP. To verify that the null hypothesis (i.e., the two treatments are equally effective in inducing IDU healing) could be true with a probability lower than 1%, and having preliminarily established for ethical reasons that the ratio between treated patients and control patients should be 2:1, we calculated (using Fisher’s exact test) that we had to enroll 30 patients to be treated with AT-G and 15 patients to be included in the control group.

Patients meeting the inclusion and exclusion criteria were consecutively enrolled in the study and randomized to receive AT-G or the SP following a sequence of randomization that consisted of a consecutive and repeated inclusion of one patient in the control group followed by two patients in the actively treated group until the achievement of the pre-established numbers of patients in the two groups had been reached.

### Statistical analysis

Demographic and clinical parameters of the patients selected in the two arms of the study were compared using an unpaired *t* test and contingency tables for continuous and categorical variables, respectively.

Data collected before and after both therapeutic procedures were analyzed using paired *t* tests and Wilcoxon tests for continuous and discrete variables, respectively.

When multiple tests were performed, we corrected the level of significance for the number of tests, according to Bonferroni’s method.

A Kaplan–Meier curve was built and the log-rank test was used to analyze the cumulative prevalence of healed IDUs in the two arms of the study in each observation time during the follow-up.

### Ethical rules

This study was conducted according to the Helsinki Declaration and approved by the local ethics committee. Written informed consent was obtained from all of the enrolled patients.

## Results

A preplanned interim analysis of the results was performed after the enrolment of 25 and 13 patients who received AT-G and the SP, respectively. The analysis demonstrated that the primary endpoint of the study was already reached (i.e., the probability that the two therapeutic procedures had no difference in efficacy for inducing IDU healing was < 1%). Considering this figure, we decided to stop the enrolment of further patients into the study.

Table 1 summarizes the main demographic and clinical characteristics of the patients included in the study. The groups of patients randomized to undergo AT-G and the SP were comparable for age, disease duration, dcSSc/lcSSc ratio, and extension of the IDU area at the time of enrollment. Patients who received active therapy had IDUs lasting for a significantly longer time than the patients from the control arm (*p* < 0.01).

**Table 1:** Demographic and clinical features in patients with SSc treated with AT-G and SP

Feature	AT-G (25 patients)	SP (13 patients)	Difference
Age (years), median (range)	42 (21–69)	37 (23–70)	n.s.
Women, *n* (%)	23 (92)	13 (100)	n.s.
Disease duration (years), median (range)	4 (1–10)	5 (2–12)	n.s.
Patients with dcSSc/lcSSc (*n*)	16/9	7/6	n.s.
IDU localization (2nd/3rd/4th/5th finger)	10/12/2/1	5/4/4/0	n.s.
IDU longest diameter, mean (95% CI)	7.5 (6.7–8.2)	7.3 (6.2–8.4)	n.s.
IDU duration before enrolment (weeks), mean (95% CI)	11.4 (9.7–13.1)	8.0 (7.1–8.8)	*p* < 0.001
Patients who have had previous ulcers, *n* (%)	15 (60)	7 (54)	n.s.

*AT-G* adipose tissue grafting, *CI* confidence interval, *dcSSc* diffuse cutaneous systemic sclerosis, *IDU* ischemic digital ulcer, *lcSSc* limited cutaneous systemic sclerosis, *n.s.* not significant, *SP* sham procedure

**Fig. 2:**
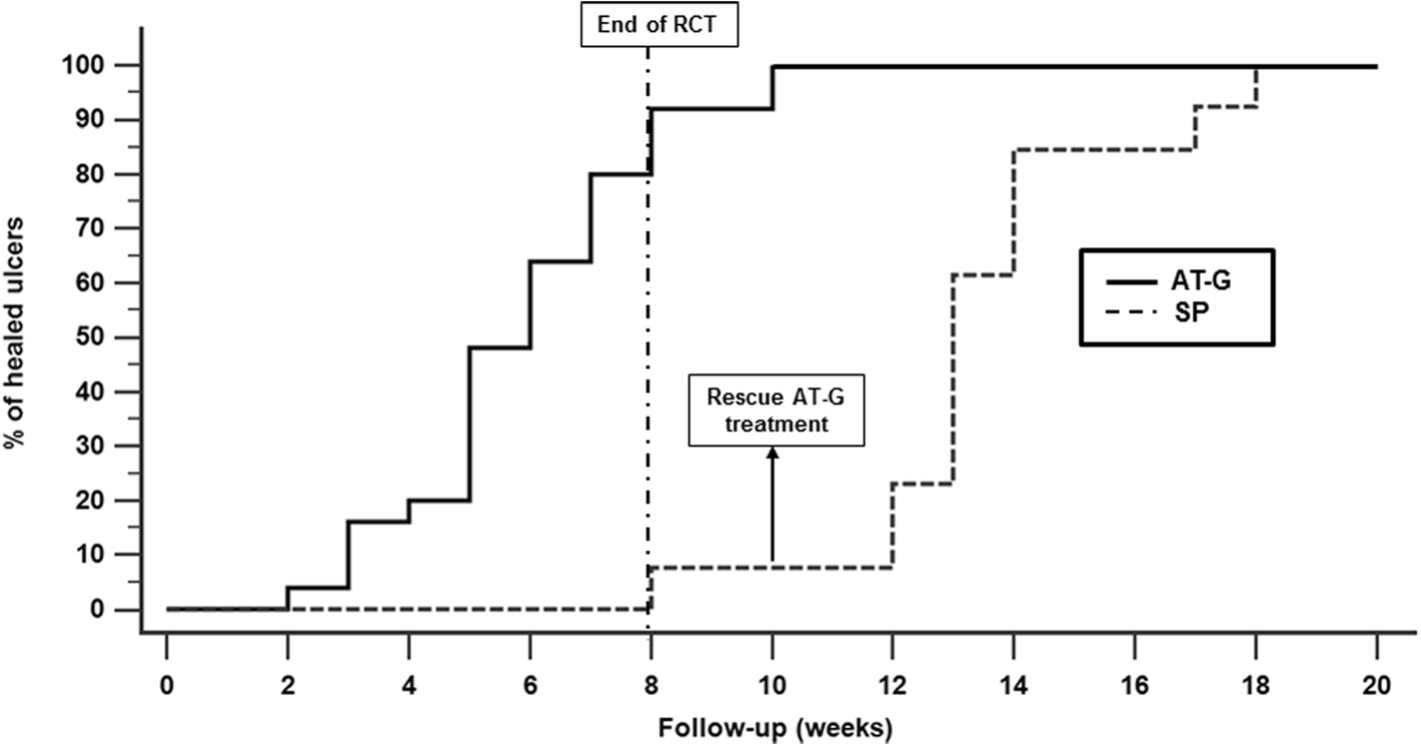
Percentages of healed IDUs in patients treated with AT-G and in those who underwent SP. During 8 weeks of RCT follow-up, number of AT-G-treated patients who achieved IDU healing was significantly higher than number of patients who underwent SP (log-rank *p* < 0.0001), with hazard ratio of 22.2 (95% CI 9.97–49.42). At 10 weeks, all 12 patients of control group whose IDU did not heal received a rescue AT-G. In following 8 weeks, DU healing was observed in all of these patients. AT-G adipose tissue grafting, RCT randomized controlled trial, SP sham procedure

**Fig. 3:**
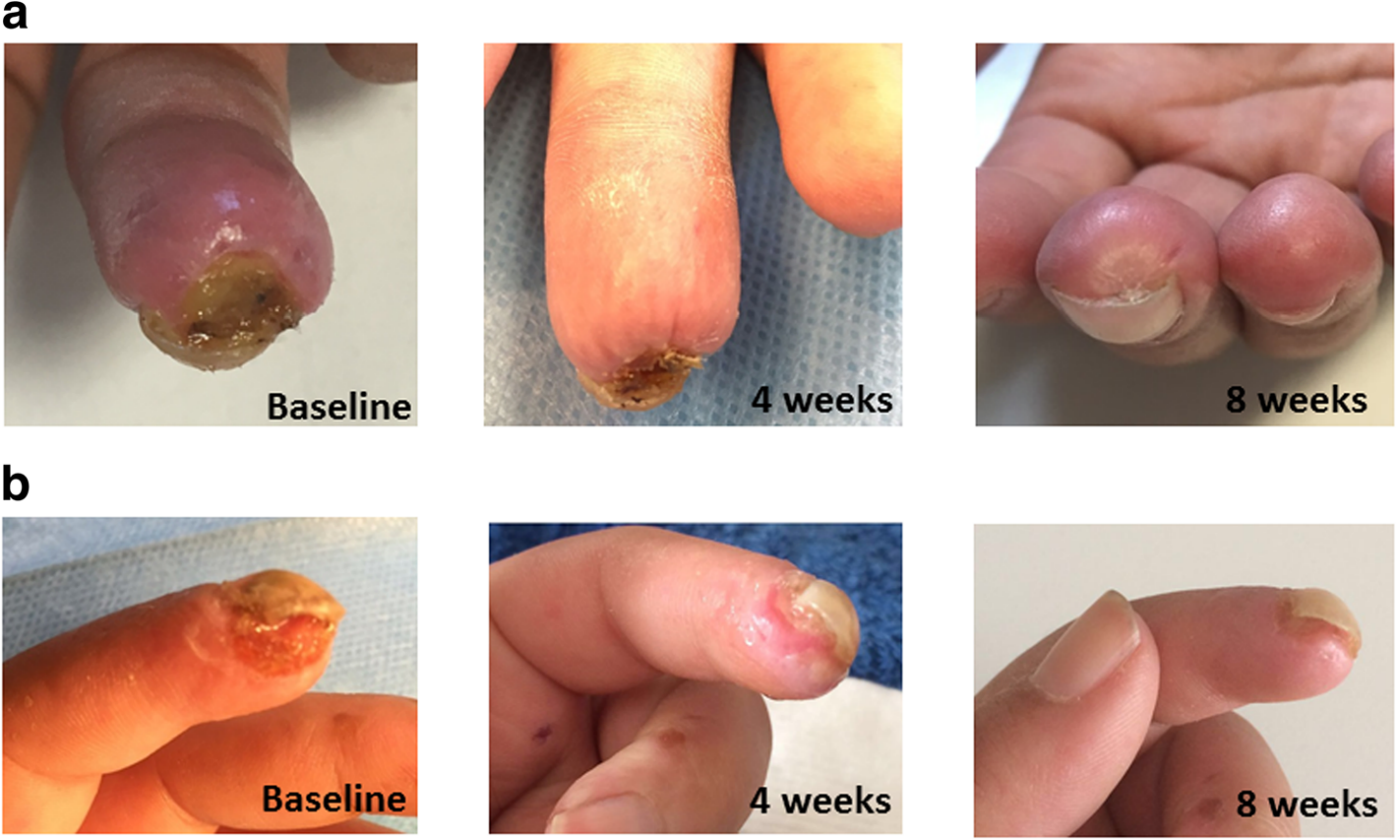
Digital ulcer progressive healing of patient 2 (**a**) and patient 8 (**b**) after autologous AT-G

Twenty-three out of the 25 patients (92.0%) treated with AT-G achieved DU healing after 8 weeks. Conversely, in only 1 of the 13 patients (7.7%) who received the SP was IDU healing observed at the end of the same observation period. This difference was highly significant (*p* < 0.0001). The Kaplan–Meier curve representing the cumulative prevalence of IDU healing in the two differently treated groups is shown in Fig. 2. Figure 3 shows digital ulcer progressive healing of patient 2 (Fig. 3a) and patient 8 (Fig. 3b) after autologous AT-G.

The result was maintained during the following 3-month period and no new DU appeared in any of the AT-G patients, both in the treated finger and the other fingers of the same hand. This feature might have been fostered by the consequent secondary prevention with bosentan.

**Fig. 4:**
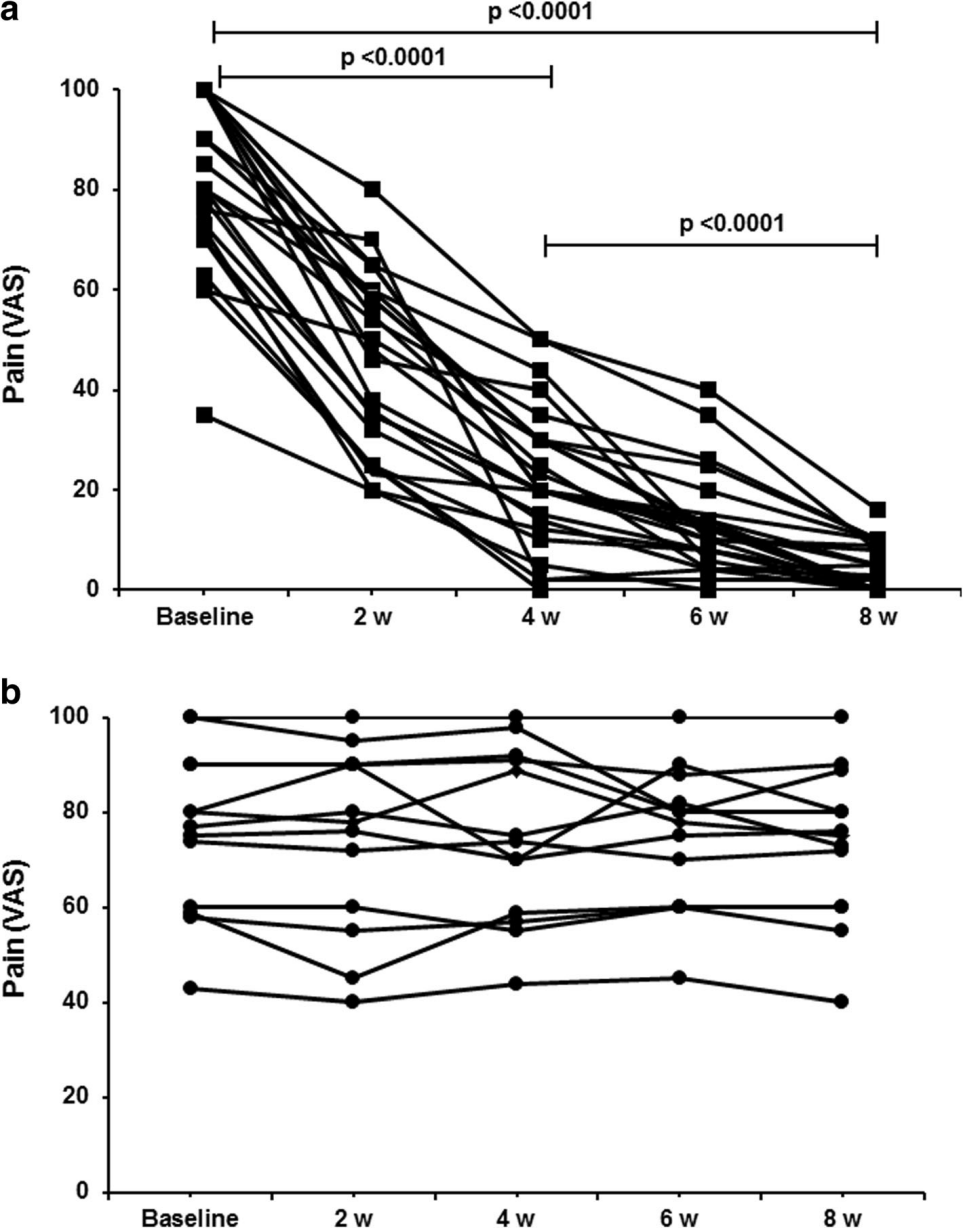
Variations of pain intensity measured by VAS in patients treated with AT-G as active therapy (**a**) and in those who underwent SP as placebo treatment (**b**). VAS for pain measured every week in all study patients. (a) Comparisons between baseline VAS values and those recorded at 4 and 8 weeks of follow-up, and between values at 4 and 8 weeks, in patients treated with AT-G are all highly significant. (b) VAS values remained unchanged in patients who underwent SP. VAS visual analog scale, w weeks

At the 4-week observation time, a reduction of the pain VAS of more than 50% of the baseline value was recorded in 21 out of the 25 patients treated with AT-G and in 0 out of the 13 patients of the SP group (*p* < 0.00001 using Fisher’s exact test). All four patients in the AT-G group in whom this level of pain improvement was not observed at the 4-week observation time reached this goal after 8 weeks. Conversely, the number of patients in the SP group with this grade of pain score reduction remained at zero even at the 8-week observation time. The variation of pain VAS within the two groups is shown in Fig. 4a, b.

**Fig. 5:**
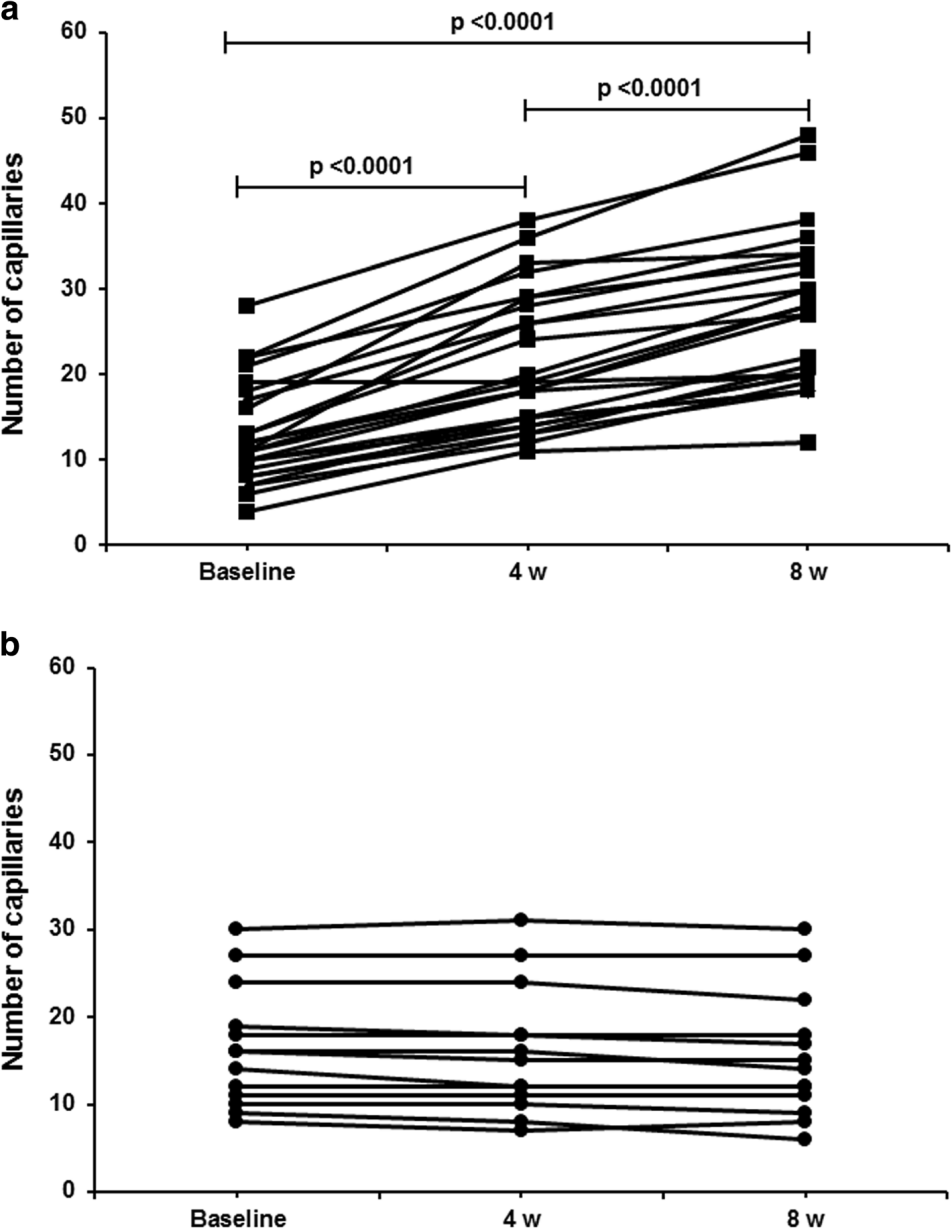
Variations in number of capillaries assessed by NVC in fingers of patients with cardinal IDU treated with AT-G as active therapy (**a**) and who underwent SP as placebo treatment (**b**). (a) Comparisons between baseline counts with those recorded at 4 and 8 weeks, and between counts recorded at 4 and 8 weeks, of follow-up in AT-G-treated patients are all highly significant. (b) Capillary counts remained unchanged in patients who underwent SP. w weeks

In the actively treated patients, the number of capillaries in the affected digit consistently increased after 4 and 8 weeks compared to the starting time and at the 8th week in comparison with 4-week counts (*p* < 0.0001 in all comparisons). Conversely, capillary counts remained unchanged in patients who underwent the SP (see Fig. 5a, b). Despite the significant capillary increase in the AT-G-treated group, patients maintained the late capillary pattern, according to Cutolo et al. [3].

It is worth noting that in all of the 12 patients who initially underwent the SP and in whom this procedure was ineffective at the end of the 10th week, IDU healing was observed in the following 8 weeks after the rescue AT-G. It is also noticeable that the two patients initially treated with AT-G and in whom the IDU healing was not achieved at the 8-week observation time had a complete resolution of their IDU at the end of the 10th week (see Fig. 2).

## Discussion

Fingertip IDUs are a frequent complication in patients with both lc-SSc and dc-SSc, and are considered a clear consequence of distal capillary bed desertification that can be observed in the late phase of the disorder [2–5]. The SSc-related ischemic DUs are often painful, invalidating, and may lead to a severe deterioration in quality of life. Their trend to healing is frequently very slow or null, despite the multiple local and general therapeutic measures that are commonly adopted [4].

This paper clearly shows that AT-G is significantly more effective than traditional vasoactive therapy alone in inducing IDU healing, restoration of the capillary bed in the treated finger, and a rapid resolution of local ischemic pain in patients with SSc. The fact that these clinical effects can be ascribed to specific actions of AT-G, and not to mechanical stimulation due to local overload of the injected material, is supported by the almost complete ineffectiveness of the SP that was blindly carried out in the control group. Furthermore, the IDUs of the patients who initially had received the ineffective SP healed within the following 8 weeks once treated with a rescue AT-G.

The results of the present RCT confirm the data we obtained in our previous open study where the same AT-G induced, after 2 months and in all of the patients, IDU healing, a significant increase in the number of fingertip capillaries, and a rapid resolution of ischemic pain [13]. Although it is likely that the local increase of the microvascular bed may be an important cause of pain reduction in the AT-G-treated patients in our study, the rapidity of this pain improvement could rather suggest the intervention of other possible factors. It has been shown in experimental models that the ASC secretome may exert either downregulation of the inflammatory cytokines or upregulation of neural trophic factors able to modulate neuroinflammation and the related pain [19–21].

Therapeutic procedures using various cellular components that derive from AT, and potentially containing totipotent stem cells, are an emerging new therapeutic approach for different disease-related features in patients with SSc [9].

Granel et al. [22] have demonstrated that local lipofilling with the separated SVF is capable of improving Raynaud’s phenomenon, local vascular flow, and skin elasticity in the hands. In another study by our group, we observed that lipofilling with autologous AT in the perioral area improves the mouth opening capacity and at the same time induces a histologically and NVC-proven new capillary formation in the local skin area [23].

In contrast, few studies, limited to single case reports, have been performed in which SSc-related ischemic lesions in fingers or limbs were treated with regional infusion of purified BM-MSCs. Even if limited in number, these experimental cell therapies have uniformly shown an improvement of ischemic lesions [7–9].

Positive results obtained with cellular therapeutic procedures with different AT fractions in SSc-related vascular lesions are not completely surprising, since similar results have been reported in other ischemic conditions in both human disorders and in animal models treated with similar therapeutic modalities. A certain degree of wound improvement and healing has been observed after regional injection of isolated ASCs in the ischemic leg in a small group of patients with lower limb ulcers that were not suitable for revascularization [24, 25]. Similarly, an acceleration of excisional wound healing by locally administered ASCs has also been demonstrated in different animal models [26, 27]. Furthermore, foot ulcers have been reported to heal more frequently and rapidly, with respect to traditionally treated controls, in diabetic patients treated locally with differently processed AT cellular fractions [24, 28].

The reason why local implantation of AT containing adipocyte progenitors, ASCs and SVF, as well as similar procedures with BM-MSCs, may induce such positive changes in different SSc-related ischemic lesions remains almost completely speculative at the moment. A number of studies have shown that ASCs, as well as BM-MSCs, are multipotent cells potentially able to differentiate themselves into other cell types, like cells of the mesoderm (chondrogenic, osteogenic, myogenic) and endoderm (endothelial) lineages, under the conditioning of different stimulant, as demonstrated in a lot of in-vitro and in-vivo studies [12]. ASCs are also capable, when locally implanted, of producing a number of growth factors with proangiogenetic and proliferation effects, such as vascular endothelial growth factor and fibroblast growth factor, that may favor local angiogenesis by exerting their paracrine actions [29]. ASCs may also directly produce or stimulate the production from other resident cell types of a number of cytokines with immune-modulating action probably capable of downregulating some pathological mechanisms in injured tissues [29]. On the other hand, damaged tissues may release various chemoattractants, like epidermal growth factor and monocyte chemoattractant protein-1, that are able to induce local migration and concentration of ASCs [12, 29] by interacting with specific receptors present on the surface of these latter cells [30]. Finally, both BM-MSCs and ASCs have shown, either in vivo or in vitro, that they better express their angiogenetic properties when exposed to hypoxic conditions [31, 32].

Since SSc is a systemic microvascular disorder where many tissue and organ systems may suffer from a reduction of capillary flow and the consequent fibrotic evolution, it could be postulated that both BM and AT may be involved in this process and the locally resident cells may be defective. This possibility could make use of stem cells of autologous origin less effective with respect to corresponding cells from a heterologous donor, when used as regenerative therapeutic elements in the treatment of SSc. Some in-vitro data suggest that isolated BM-MSCs have a senescent aspect and may be defective in their immunosuppressive properties [33]. Conversely, studies assessing the functional capacity of ASCs from SSc patients in comparison to those of healthy controls have shown that the proliferative and proangiogenetic ability of these cells is not compromised, probably being preserved in the AT niche from any SSc-related pathological aggression [34].

The SVF of the medium layer of AT separated by centrifugation contains a heterogeneous population of many cells other than ASCs, including adipocyte precursors, endothelial cells and their progenitors, pericytes, hematopoietic lineage cells, and fibroblasts. At the present time, little information is available on the possible contribution given by the SVF in the regenerative effects that whole AT has shown under many different conditions [35]. Recent studies have demonstrated that pericytes possibly provide a very important contribution to tissue repair and revascularization, since these cells may work as driving stromal cells in tubuli formation during angiogenesis [36]. Therefore, it cannot be excluded that SVF, where pericytes are certainly present, might play an important role also in the healing process of IDUs in our patients. Such an assumption is further reinforced by recent data showing the higher angiogenetic potential of freshly isolated AT where the SVF is largely represented, when compared to cultured AT cells where the SVF is reduced [12].

The multiple lineage differentiation potential of ASCs, the possible support to angiogenesis given by the SVF, and the local release of a number of growth factors and cytokines, able to stimulate the angiogenesis, may all have contributed to the formation of new capillary loops, and to the rapid IDU healing observed in our study.

The present study confirms previous more preliminary and uncontrolled data suggesting that local transfer of autologous AT may be a successful option to induce healing in ischemic SSc-related fingertip IDUs that are resistant to more traditional therapeutic approaches. The AT-G procedure we have adopted in this study did not cause any adverse event and so it has been proven to be a safe treatment. Since this procedure is fairly invasive, at the present time it should be considered a second-line treatment to be carried out only in cases resistant to traditional therapy. Only the acquisition of experience in this procedure, followed by a careful analysis of its cost/effectiveness balance, could change present behavior.

## Conclusions

This study clearly shows that AT-G is significantly more effective than traditional vasoactive therapy alone in inducing IDU healing, restoration of the capillary bed in the treated finger, and a rapid resolution of local ischemic pain in patients with SSc. No adverse event was registered during the study. Altogether, our data suggest that AT-G may be considered a successful option to induce healing in ischemic SSc-related IDUs.

## Abbreviations

ASC: Adipose-derived stromal cell
AT: Adipose tissue
BM-MSC: Bone marrow mesenchymal stem cell
dc: Diffuse cutaneous
G: Grafting
IDU: Ischemic digital ulcer
lc: Limited cutaneous
NVC: Nailfold videocapillaroscopy
SP: Sham procedure
SSc: Systemic sclerosis
SVF: Stromal/vascular fraction
VAS: Visual analogue scale
